# Flow diversion treatment for giant intracranial serpentine aneurysms

**DOI:** 10.3389/fnagi.2022.988411

**Published:** 2022-11-03

**Authors:** Xin Tong, Zijun He, Mingyang Han, Xin Feng, Chuanzhi Duan, Aihua Liu

**Affiliations:** ^1^Neurointerventional Center, Beijing Neurosurgical Institute and Beijing Tiantan Hospital, Capital University, Beijing, China; ^2^Department of Neurosurgery, The Third Xiangya Hospital, Central South University, Changsha, Hunan, China; ^3^Neurosurgery Center, Department of Cerebrovascular Surgery, Engineering Technology Research Center of Education Ministry of China on Diagnosis and Treatment of Cerebrovascular Disease, Zhujiang Hospital, Southern Medical University, Guangdong, China; ^4^Guangdong Provincial Key Laboratory on Brain Function Repair and Regeneration, Guangdong, China

**Keywords:** giant serpentine aneurysm, flow diversion, angiographic outcome, clinical outcome, complication

## Abstract

**Background:**

Giant serpentine aneurysms (GSAs) are among the most complex and challenging type of intracranial aneurysms. Surgical clipping, bypass, or endovascular parent artery occlusion has been the main treatment of GSAs in the past. However, studies on flow diversion (FD) are limited. Therefore, we reported our experience with patients with GSAs treated with FD.

**Methods:**

Patients with GSAs treated with FD from 2012 to 2020 in our single center were retrospectively reviewed. Angiographic outcomes were graded according to the O’Kelly–Marotta scale as complete occlusion (D), trace filling (C), entry remnant (B), or aneurysm filling (A). Clinical outcomes were assessed using the modified Rankin scale (mRS) score. We also collected the patients’ treatment details and perioperative complications.

**Results:**

Thirteen patients with 14 aneurysms were included, including three in the anterior circulation and 11 in the posterior circulation. Grades B–D were found in 72.7% (8/11) of the GSAs. Good prognosis (mRS score, 0–2) was found in 66.7% (8/12) and 50.0% (6/12) of the patients at the 6-month and latest follow-up, respectively. Parent artery occlusion was found in three cases of GSAs. Five postoperative complications were observed, including two minor complications and three major complications.

**Conclusion:**

Although reconstructive treatment with FD could be considered as one of the treatment strategies for patients with both anterior and posterior circulation GSAs, however, the risk of complications and parent artery occlusion should be considered.

## Introduction

Giant serpentine aneurysms (GSAs) are a rare but complex subtype of intracranial aneurysms, accounting for 17.6% of giant aneurysms and <0.1% of all aneurysms (Xu et al., 2016). The distinctive features of this type of aneurysm are a twisted parent artery with separate inflow and outflow tracts; a long lesion range (>25 mm), partially thrombosed; and a “serpiginous-like” morphology (Segal and McLaurin, 1977). However, the pathogenesis of these aneurysms remains unclear. A previous study has reported that GAS might be caused by continuous expansion (“Coandă effect”) of saccular aneurysm (Fodstad et al., 1978), originating from fusiform/dolichoectatic aneurysm (Anson et al., 1996; Senbokuya et al., 2012), and is associated with abnormalities in the blood vessel wall (Bakaç et al., 1997). Dynamic thrombosis may also play a vital role in the pathophysiology of GSA formation (Kandemirli et al., 2018).

The aims of GSA treatment are to maintain distal circulation, halt the growth of aneurysms, and decrease the mass effect. In previous studies, treatment options for GSA were limited and included direct resection with or without revascularization and bypass combined with occlusion of the inflow or outflow pathways. Endovascular treatments, such as balloon or coiling occlusion of the parent artery, have been reported as alternative options to GSA treatment (Oh et al., 2016; Kandemirli et al., 2018; Deng and Feng, 2019; Dao et al., 2021; Mizuno et al., 2021; Civlan et al., 2022). However, simply occluding the GSA without ensuing adequate circulation to the distal brain parenchyma may not be consistent with the pathological characteristics of GSA, especially in the blood vessels that supply blood to critical areas of the brain.

More recently, flow diversion (FD) devices, such as the Pipeline Embolization Device (PED; Medtronic, Minneapolis, Minnesota, United States) or the Tubridge Embolization Device (TED; MicroPort NeuroTech, Shanghai, China), have been used to treat complex and giant aneurysms (Deng and Feng, 2019). Theoretically, compared with direct resection or coiling/balloon parent artery occlusion, FD devices can reconstruct the vessels included in GSA lesions, thus maintaining distal circulation. However, this technology has been used in only a few studies with a limited number of patients. Here, we reported our experience with patients with GSA treated with FD devices.

## Materials and methods

### Study population

This study was approved by the Institutional Review Board of Beijing Tiantan Hospital. All patients treated with FD devices, including PED and TED, from Beijing Tiantan Hospital were retrospectively reviewed to identify patients with GSA from January 1, 2013, to December 31, 2020. Baseline patient and aneurysm information was collected from medical records and cerebral angiograms. In general, patients with previous treatment or insufficient data were excluded.

### Endovascular procedure

All treatments were performed under general anesthesia *via* a transfemoral approach, and systemic heparinization was administered after sheath placement. For all procedures, a 6F, 90-cm NeuroMax long sheath (Penumbra, United States) was used in the C1–C2 or V2 segments. By the navigation of a 0.014 Synchro microwire, the FD deployment microcatheter was passed through the aneurysm with the aid of a distal access catheter (5F or 6F) in the proximal parent artery. In general, we used a co-axial catheter platform system in single FD treatment and used a tri-axial platform system in FD assisted coiling treatment. If the aneurysm had a high risk of rupture, detachable coils was used to embolize it. Aneurysms are considered to be at high risk of rupture when the aneurysm is large, or has irregular morphology, or has a direct blood inflow (for example, the outside of the siphon bend). We do not consider the application of coils when the parent artery is relatively straight, or when most of the aneurysm body is located in the inwards of the parent artery curve or when the aneurysm morphology is smooth and regular. If one flow diverter device was insufficiently long to cover the entire aneurysm, another flow diverter was employed. Under such circumstances, the FD deployment microcatheter passes through the former FD and then delivers another FD to completely cover the aneurysm. After the procedure, we selected the O’Kelly–Marotta grading scale as the standard criterion for evaluating immediate angiographic outcomes. Occlusion was graded according to the O’Kelly–Marotta scale as complete occlusion (D), trace filling (C), entry remnant (B), or aneurysm filling (A) (O’kelly et al., 2010). Complete occlusion was defined as grade D, and incomplete occlusion as grades A–C. We also defined grades B–D as successfully halting the growth of GSA. Imaging evaluation was conducted by a special group composed of experienced neuroradiologists. The first follow-up of the patients was performed 3–6 months after the treatment. Latter follow-ups were performed every 12 months for incomplete occlusion aneurysms and not regularly performed for complete occlusion aneurysms. We only collected the patients’ clinical outcomes through telephone inquiries for patients without hospitalization or outpatient follow-up.

### Antiplatelet and anticoagulation treatment

All patients were premedicated with antiplatelet drugs for at least 5 days before the intervention. The patients received 75 mg of clopidogrel and 100 mg of aspirin daily. During the procedures, all patients were systemically heparinized with an activated clotting time of ≥200 s. Dual antiplatelet therapy (75 mg of clopidogrel and 100 mg of aspirin daily) was continued for at least 6 months after the procedure. Patients who were identified as clopidogrel non-responders were administered aspirin (100 mg daily) and ticagrelor (90 mg twice daily). Aspirin was continued for life according to the standard embolism prophylaxis for intraluminal stent placement.

## Results

### Baseline information

During the study period, 908 patients with 1,100 aneurysms treated with FD devices were reviewed, and 13 patients with 14 aneurysms were diagnosed with GSA (Table 1). The median age of the patients with GSA was 60 (range, 11–72) years, and 84.6% of the patients (11/13) were male. The clinical manifestations of the patients with GSA included headache, hemiparesis, subarachnoid hemorrhage (SAH), blurred vision, and loss of consciousness. Most aneurysms were located at post-circulation (78.6%, 11/14), including six basilar artery (BA) aneurysms (42.9%), one left vertebral artery (VA) aneurysm (7.1%), one right VA aneurysm (7.1%), and three vertebrobasilar junction (VBJ) aneurysms (21.4%). Angiographic follow-up data were obtained from 11 aneurysms, and clinical follow-up data were collected for 12 patients. The middle last angiographic and clinical times were 10 (range, 2–65) and 33 (range, 18–66) months, respectively. No clopidogrel non-responders was found in enrolled patients.

**Table 1:** Clinical data of all included patients.

No.	Clinical presentations	Site	Size	Stent type/model	Coiling	Angiographic FU/duration	Postoperative complications	Preoperative mRS	6-month FU mRS	Latest FU mRS
1	Intermittent headache	RICA	17 mm × 28 mm	PED400-30, PED400-30	Y	D/46 M	N	1	0	0/46 M
2–1	No vision in left eye	RICA	12 mm × 26 mm	PED475-35	N	PAO/65 M	N	2	3	6/67 M
2–2		LICA	21 mm × 101 mm	Placement Failed	N	PAO/59 M	N			
3	Bilateral blurred vision	VBJ	17 mm × 28 mm	PED425-35 (Balloon)	Y	D/10 M	N	1	0	6/12 M
4	Subarachnoid hemorrhage	VBJ	9 mm × 34 mm	PED350-35, PED350-35	Y	PAO/2 M	N	2	0	0/5 M
5	Intermittent headache, mild right hemiparesis	BA	18 mm × 32 mm	TED650-45, TED650-35	Y	C/4 M	N	3	2	6/38 M
6	Dizzy	BA	18 mm × 30 mm	PED450-35	N	D/20 M	Thrombotic event (right abducens nerve palsy)	1	2	2/34 M
7	Mild right hemiparesis	BA	10 mm × 28 mm	PED375-35, PED375-35	N	C/13 M	N	1	0	0/33 M
8	Progressive left hemiparesis, loss of consciousness	BA	11 mm × 66 mm	TED450-55, TED450-65	Y	NA	Subarachnoid hemorrhage	5	Death in hospital	Death in hospital
9	Persistent headache	VBJ	20 mm × 29 mm	PED325-35, PED325-30	N	D/4 M	N	1	0	0/32 M
10	Weakness of both lower extremities	BA	14 mm × 26 mm	TED450-40	N	NA	Thrombotic event (progressive right hemiparesis, loss of consciousness)	3	5	5/29 M
11	Progressive right hemiparesis, loss of consciousness	LVA	8 mm × 45 mm	PED375-35, PED350-25	N	NA	Thrombotic event (progressive loss of consciousness)	5	2	1/24 M
12	Dizzy	BA	13 mm × 32 m	PED500-35, PED450-35	Y	C/7 M	Thrombotic event (progressive right hemiparesis)	1	3	3/20 M
13	Persistent headache	RVA	12 mm × 37 mm	TED450-45, TED450-40	N	C/6 M	N	1	Lost to follow-up	Lost to follow-up

FU, follow-up; mRS, modified Rankin Scale; PAO, parent artery occlusion; RICA, right internal carotid artery; LICA, left internal carotid artery; VBJ, vertebrobasilar junction; BA, basilar artery; LVA, left vertebral artery; RVA, right vertebral artery.

### Angiographic and clinical outcomes

The FD device was successfully deployed in 13 aneurysms. One aneurysm (case 2–2) had only a PED deployed at the distal end of the GSA. However, other FD devices cannot be successfully placed owing to the extremely twisted morphology of the parent vessel. Among the successfully treated 13 GSAs, 69.2% (9/13) were treated with multi-FD devices and 30.7% (4/13) were treated with TED. In the latest angiographic follow-up, parent artery, complete (grade D), and incomplete occlusions were found in 27.3% (3/11), 27.3 (3/11), and 45.5% (5/11) of GSAs, respectively. However, all incomplete occlusion aneurysms had achieved a grade B or C; thus, they could also be considered successful in halting the growth of GSAs.

Postoperative complications were observed in 38.5% (5/13) of GSA patients, including one SAH, two transient ischemic events, and two sustained ischemic events. Improvement of clinical manifestation and good prognosis (modified Rankin Scale [mRS] score, 0–2) were found in 66.7% (8/12, one lost to follow-up) of the patients at the 6-month follow-up after the procedure. In the latest follow-up, good prognosis was found in 50.5% (6/12) of the patients, and the all-cause mortality rate was 33.3% (4/12). Compared with preoperative status, the mRS dropped in 7 patients (58.3%, 7/12) at 6-month follow-up and in 5 patients (41.7%, 5/12). One patient died in the hospital because of GSA rupture. One patient with bilateral internal carotid artery (ICA) GSAs treated with an FD device developed bilateral ICA occlusion and died after 5 years. One patient refused to take antiplatelet drugs after 3 months and eventually died because of a devastating ischemic stroke.

### Case presentations

#### Case presentation 1

**Figure 1:**
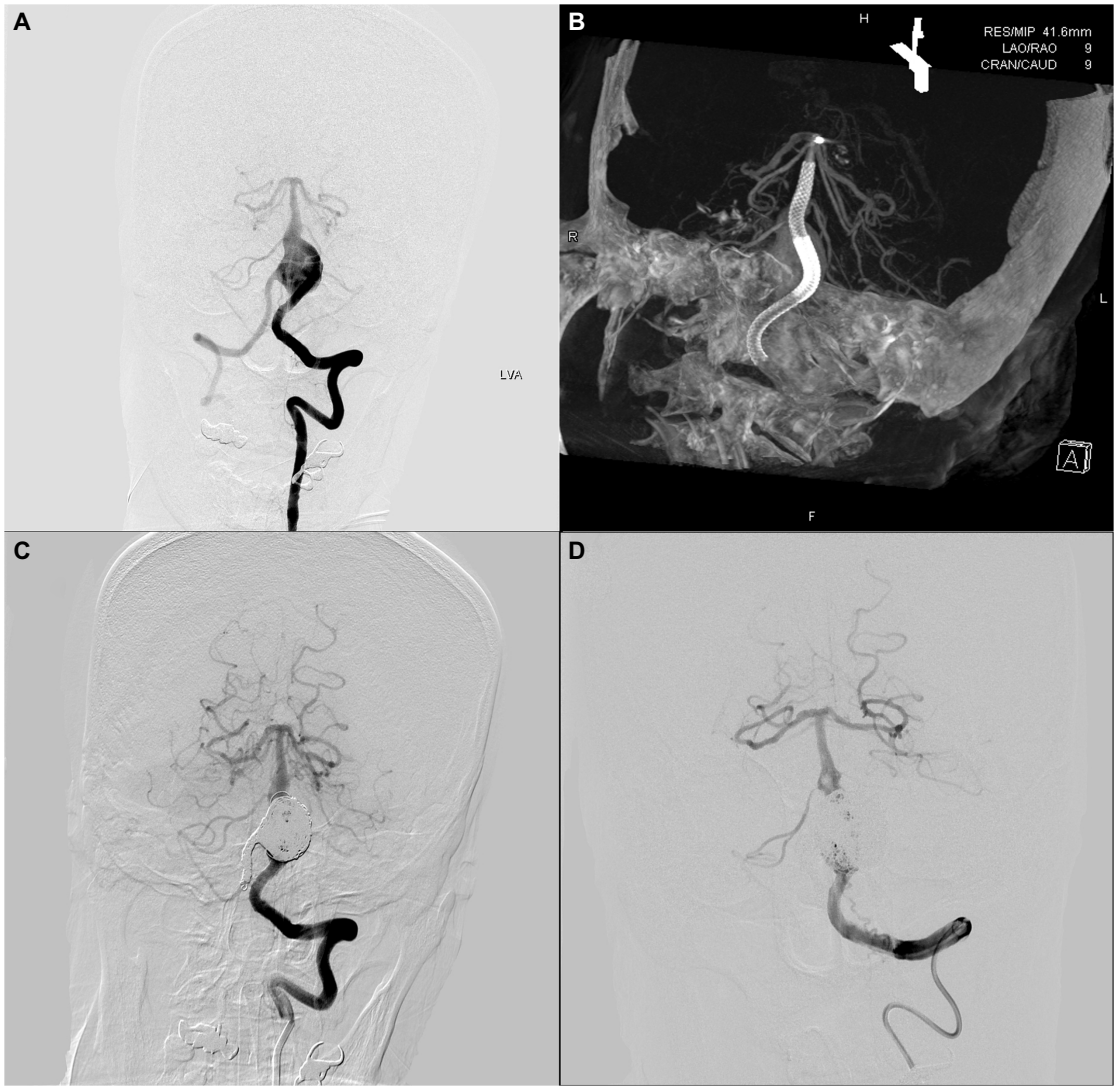
An adult patient with a 20 mm × 29 mm vertebrobasilar artery junction serpentine aneurysm. The preoperative, intraoperative, immediate postoperative, and latest follow-up angiography images are shown in **A–D**.

An adult patient was admitted to our center because of a persistent headache. Cerebral angiography revealed a vertebrobasilar artery serpentine aneurysm with a diameter of 20 × 29 mm (Figure 1A). Subsequently, this patient underwent double-PED bridge deployment in the left vertebral and basilar arteries with a balloon occluding the V4 segment of the right VA (Figures 1B,C). Four months later, the patient underwent cerebral angiography, which showed that the serpentine aneurysm was totally occluded (Figure 1D).

#### Case presentation 2

**Figure 2:**
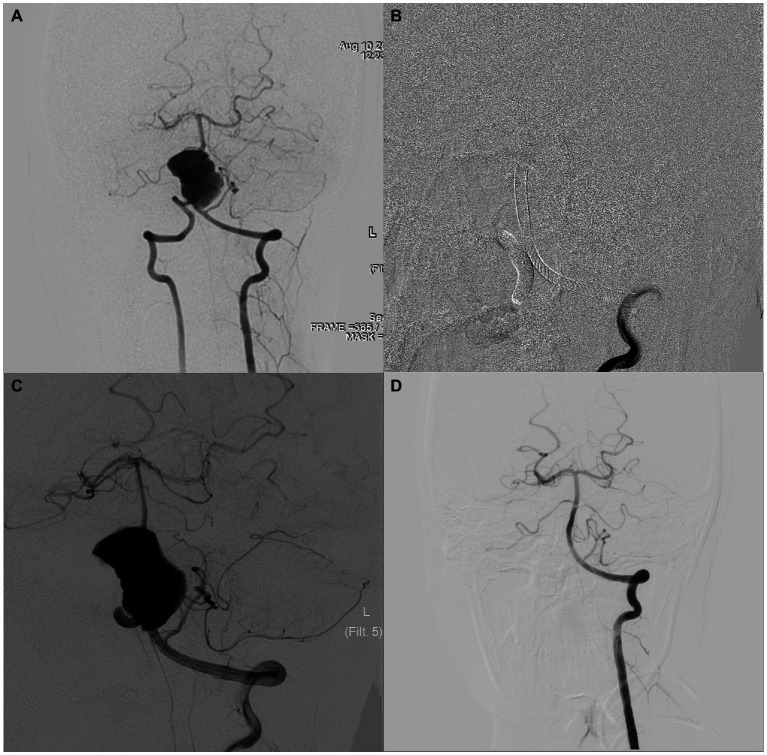
An adult patient with a 17 mm × 28 mm vertebrobasilar artery serpentine aneurysm. The preoperative, intraoperative, immediate postoperative, and latest follow-up angiography images are shown in **A–D**.

An adult patient presented with a mass on noncontract computed tomography, and computed tomography angiography revealed a vertebrobasilar artery giant aneurysm. Cerebral angiography revealed that the aneurysm was approximately 17 mm × 28 mm in size (Figure 2A). This patient underwent double-PED bridge deployment in the left vertebral and basilar arteries plus coil embolization of the aneurysm cavity and V4 segment of the right VA (Figures 2B,C). Ten months later, cerebral angiography was reviewed, which showed that the aneurysm had a subtotal occlusion (Figure 2D).

## Discussion

The treatment of GSA is challenging owing to its complex imaging and clinical features. GSA refers to large (>25 mm) aneurysms with separate inflow and outflow tracts and partial thrombosis. On the one hand, treatment of GSA must halt the growth of aneurysms and decrease the mass effect; on the other hand, treatment of GSA must carefully evaluate the circulation to supply sufficient blood to the distal brain parenchyma. In previous studies, craniotomy and parent artery occlusion with coiling or balloon have been the primary treatment approaches for GSA. However, few studies have reported the outcomes of GSA in patients treated with an FD device. In this study, we reported our experience with this technology with the largest study population, to the best of our knowledge.

In general, GSA was more common in young men than in young women and in the anterior circulation than in the posterior circulation according to a previous study (Kandemirli et al., 2018). This finding is partially like our data, which showed that the middle age was 60 years, and the percentage of men was 81.4%. However, most GSAs in this study were located in the posterior circulation, including the BA, VA, and VBJ. This difference may be because posterior circulation aneurysms can be challenging to treat with craniotomy or parent artery occlusion.

In previous studies, surgical resection was typically used to treat GSAs (Xu et al., 2016). Recently, endovascular occlusion has been successful in treating GSAs (Oh et al., 2016; Kandemirli et al., 2018; Deng and Feng, 2019; Dao et al., 2021; Mizuno et al., 2021; Civlan et al., 2022). Kandemirli et al. reported 15 patients from three centers who underwent aneurysm and parent artery occlusion with coiling or balloon, and improvement or resolution of symptoms without any treatment-related morbidity was achieved in 11 patients (Kandemirli et al., 2018). Dao et al. reported on 22 patients who underwent selective treatment with parent artery occlusion at the site of GSAs, with no clinical complications, and none of the patients had recurrence after selective embolization (Dao et al., 2021). Preserving the parent vessels is one of the most essential objectives for treating GSAs. However, it is difficult to perform traditional endovascular treatment, such as single or stent-assisted coiling, to achieve this purpose because there is no definable neck (Kandemirli et al., 2018; Dao et al., 2021). The favorable outcome obtained in these studies is possibly the location of GSA, which has rich collateral circulation, such as the P2 segment, and thus can be directly occluded. However, most of the GSAs in our study were located in the main vessels of the posterior circulation, which may be considered “treatment-restricted areas” with previous approaches. In the last follow-up, 72.7% of GSAs had achieved grade C or D and had stopped the growth of GSAs. One of the advantages of FD is that it can reconstruct the parent artery and decrease the parent artery tortuosity (Waihrich et al., 2018). Therefore, the inflow and outflow tracts of GSAs can still be maintained to supply sufficient blood to the distal area. Meanwhile, compared with clipping or coiling, FD showed more advantages in decreasing the volume of aneurysms and thus can be more effective in improving the mass effect (Carneiro et al., 2014; Miyachi et al., 2017; Silva et al., 2018; Wang et al., 2019).

Compared with other types of aneurysms, FD for GSAs has unique characteristics. First, multi-FD devices were more common in GSA because of their large lesion nature. In our study, 69.2% (9/13) of the GSAs were treated with multi-FD devices. Generally, multi-FD devices may prolong the procedure time and increase the difficulty of the operation (Tan et al., 2015). Young et al. reported a multiple PED twisting phenomenon during treatment of a large fusiform aneurysm (Young et al., 2019). The complex and twisted lesions of GSA can further increase the difficulty of microcatheter placement, stent deployment, and stent bridging. In our study, one patient encountered this situation, which may have indirectly resulted in parent artery occlusion. The clinical outcomes of patients treated with this technology have been reported to have conflicting results in previous studies. Link et al. reported that multiple PEDs improved aneurysm occlusion without increasing morbidity in a single study with 140 patients (Link et al., 2021). However, Tan et al. found that a longer procedure time and multiple PEDs were risk factors for symptomatic thromboembolic events (Tan et al., 2015). In our study, satisfactory angiographic outcomes (grade C or D) were found in 50% (2/4, one was excluded due to lost to follow-up) of the single FD group and 85.7% (6/7) of the multi-FD group. However, the rates of postoperative complications were 40% (2/5) and 33.3% (3/9) in the single FD and multi-FD groups, respectively. This result may indicate that multi-FD device use can successfully improve the angiographic result without increasing complication morbidities.

Second, although multi-FD device use can cover the neck of GSAs as much as possible, there may still be remnants of the neck, especially in particular locations. In this study, one patient with GCS lesion from the top of the BA to the upper end of the VBJ had been only treated with multi-PED from beginning to end of the BA after careful discussion by a group of neurointerventional experts. The GCS lesion at the top of the BA was relatively small and not covered with PED and thus had no changes in this part at the latest angiographic follow-up, whereas the other part covered with PEDs achieved grade D. Although growth was not observed at follow-up, the risk of growth or rupture of the untreated part of the GSA still exists.

Third, although one of the greatest advantages of FD is the preservation of the original parent artery of GSAs, there are still risks for patients to develop parent artery occlusion. In this study, three lesions observed in two patients were finally diagnosed with parent artery occlusion at the latest follow-up. One patient had VBJ occlusion, but without any clinical symptoms. This was because the VAs was still retained at V3–V4 and the anterior circulation was well compensated for by the superior BA. However, another patient with bilateral ICA occlusion died of progressive clinical manifestation. The reason for the parent occlusion remained unknown (Potts et al., 2017). Several factors may affect the occlusion of the parent vessel after PED placement, such as the intrinsic thrombogenicity of the endoluminal construct, dosing and drug resistance of antiplatelet therapy, coagulopathic disposition of the patient, and degree of stenosis of the parent vessel. The large, twisted, and “serpiginous-like” morphology and partially thrombosed nature of GSAs may increase the risk of parent artery occlusion. However, the causal relationship between these characteristics and outcome cannot be clarified in our study. Although there is a risk of occlusion, FD may still be the best choice, considering the difficulty of implementing other treatments in the BA and VA. Compared with direct occlusion of the BA or VA, chronic occlusion caused by FD may result in a higher adaptation time. From this perspective, FD may be beneficial to patients.

In the 14 GSAs produced in this study, five postoperative complications were observed, including two minor complications and three major complications. Four complications occurred in the BA GSAs and one in the left VA GSA. Importantly, all major complications occurred in BA GSAs. These results differ from those of previous studies that included patients with endovascular parent occlusions. The primary reason for this difference may be that most GSAs reported in previous studies were located in the middle or posterior cerebral artery. However, GSAs related to the BA, VA, or VBJ have rarely been reported and theoretically have more challenges. Among the three major complications, one patient with progressive left hemiparesis and consciousness disorder was diagnosed with BA GSA during emergency rescue treatment and developed SAH while undergoing FD treatment, whereas the other two patients developed hemiparesis after the procedure (Alwakeal et al., 2021). Previous studies have reported an overall mortality rate of 10.6%, with major complications observed in 22%, angiographic occlusion in 65%, and good functional outcomes in 67% of the patients with posterior circulation aneurysms treated with FD^23^. Generally, patients with VBJ and BA aneurysms have poor natural histories (Kobayashi et al., 2014; Saliou et al., 2015) and pose significant treatment challenges regardless of the treatment technique (Sanai et al., 2008; Saliou et al., 2015; Sönmez et al., 2015), especially in patients with GSAs. Although an FD device is an important tool in the treatment of GSAs within both the anterior and posterior circulations, the ultimate choice of treatment technique must thoroughly consider the individual anatomical, patient, and treating physician factors.

### Limitations

Our study has some limitations. First, the retrospective design and *post hoc* analysis may have introduced selection bias. Meanwhile, angiographic follow-up was lost in three (21.4%) patients, and clinical follow-up was lost in one (7.7%) patient. Second, thorough analyses of the outcomes were difficult to perform because of the limited population in this study; however, to the best of our knowledge, this is the largest study of patients with GSA treated with FD to date. Finally, the single-center nature of this study may hinder the generalization of the results. Further studies of GSA treated with FD are necessary to investigate the safety and efficacy of this technology.

## Conclusion

Reconstructive treatment with FD could be considered as one of the treatment strategies for patients with both anterior and posterior circulation GSAs; however, the risks of complications and parent artery occlusion should be considered.

## Data availability statement

The raw data supporting the conclusions of this article will be made available by the authors, without undue reservation.

## Ethics statement

The studies involving human participants were reviewed and approved by institutional research ethics boards of Beijing Tiantan Hospital. The patients/participants provided their written informed consent to participate in this study.

## Author contributions

AL and CD conceived and designed the study. XF and MH collected the data. XT and ZH conceived of the project, analyzed the data, and wrote the paper. XF reversed the manuscript. All authors read and approved the final manuscript.

## Funding

This work was supported by the Natural Science Foundation of China (81771233, 82171290); Research and Promotion Program of Appropriate Techniques for Intervention of Chinese High-risk Stroke People (GN-2020R0007); BTH Coordinated Development-Beijing Science and Technology Planning Project (Z181100009618035); Beijing Municipal Administration of Hospitals’ Ascent Plan (DFL20190501) and Beijing Natural Science Foundation (19L2013, 22G10396).

## Conflict of interest

The authors declare that the research was conducted in the absence of any commercial or financial relationships that could be construed as a potential conflict of interest.

## Publisher’s note

All claims expressed in this article are solely those of the authors and do not necessarily represent those of their affiliated organizations, or those of the publisher, the editors and the reviewers. Any product that may be evaluated in this article, or claim that may be made by its manufacturer, is not guaranteed or endorsed by the publisher.
